# Genome-Wide Identification of *Aqp* Family Related to Spermatogenesis in Turbot (*Scophthalmus maximus*)

**DOI:** 10.3390/ijms241411770

**Published:** 2023-07-21

**Authors:** Xueying Wang, Ning Zhao, Tao Wang, Jinwei Huang, Qinghua Liu, Jun Li

**Affiliations:** 1 CAS and Shandong Province Key Laboratory of Experimental Marine Biology, Institute of Oceanology, Chinese Academy of Sciences, Qingdao 266071, China; xueyingwang@qdio.ac.cn (X.W.);; 2 Laboratory for Marine Biology and Biotechnology, Qingdao National Laboratory for Marine Science and Technology, Qingdao 266237, China; 3 University of Chinese Academy of Sciences, Beijing 100049, China; 4 School of Marine Science and Engineering, Qingdao Agricultural University, Qingdao 266109, China

**Keywords:** turbot, *aqps*, spermatogenesis, sperm

## Abstract

The development and maturation of sperm entails intricate metabolic processes involving water molecules, amino acids, hormones, and various substances. Among these processes, the role of aquaporins (*aqps*) in the testis is crucial. Turbot (*Scophthalmus maximus*) is a significant marine flatfish species in China; however, natural egg laying in females is not feasible under cultured conditions. Consequently, artificial insemination becomes necessary, requiring the retrieval of sperm and eggs through artificial methods. In this study, we combined genomic, transcriptomics, RT-qPCR, computer-assisted sperm analysis (CASA), and immunohistochemistry to investigate the involvement of the *aqp* family in spermatogenesis in turbot. Through genomic data analysis, we identified 16 *aqps* genes dispersed across 13 chromosomes, each exhibiting the characteristic major intrinsic protein (MIP) domain associated with AQPs. The results from RNA-seq and RT-qPCR analysis revealed prominent expression of *aqp4*, *10*, and *12* during the proliferative stage, whereas *aqp1* showed primary expression during the mature stage. *aqp11* displayed high expression levels during both MSII and MSV stages, potentially contributing significantly to the proliferation and maturation of male germ cells. Conversely, *aqp8* showed elevated expression levels during the MSIII, MSIII-IV, and MSIV stages, suggesting its direct involvement in spermiogenesis. Immunohistochemical analysis unveiled the predominant localization of AQP1 protein in male germ cells rather than Sertoli cells, specifically concentrated in the head of sperm within cysts. Furthermore, a noteworthy decline in sperm motility was observed when sperm were subjected to treatment with either the AQP1-specific inhibitor (HgCl_2_) or the AQP1 antibody. However, no direct correlation was found between the expression of *Smaqp1* and sperm quality. Overall, these findings provide new insights into the involvement of *aqps* in teleost spermatogenesis. Moreover, they hold potential for improving techniques related to sperm activation and cryopreservation, offering valuable knowledge for future advancements in this field.

## 1. Introduction

The maintenance of homeosmotic balance is crucial for the survival and proper functioning of both marine and freshwater teleosts [1,2]. Aquaporins (*aqps*), a family of small, hydrophobic integral membrane channel proteins, play a vital role in regulating the movement of water and ions across cellular membranes by facilitating their transport along osmotic gradients [3,4]. Water homeostasis is a critical factor during gametogenesis and reproduction. Spermatogenesis, involving the maturation, concentration, and storage of sperm in seminiferous vessels is intricately connected to substantial fluid secretion and absorption within the male reproductive tract [5,6,7]. Several aquaporins have been identified in the male reproductive systems, and they are thought to play a role in mediating many of these physiological processes [8,9,10]. Aquaporins are involved in the process with complex metabolism of water molecules, amino acids, hormones, and other substances. However, the expression and function of aquaporins during spermatogenesis are still poorly understood, and limited research has been conducted in this area [3,11]. In prior studies, *Aqp0* was found to be localized to Sertoli cells; *aqp1* exhibited expression in germ cells, particularly in sperm. *Aqp7* and *aqp8* were implicated in spermatid differentiation to sperm, while *aqp11* was involved in cytoplasmic recycling and maintenance of Sertoli cells [9,12,13,14,15,16].

Recently, there has been a growing focus on the significant role of aquaporins in the maturation of male germ cells in marine teleosts. Within the testis and vas deferens, teleost sperm remains dormant, only transitioning into an active state upon release into the aquatic environment. Notably, the pivotal factor influencing sperm activation is osmolality [1]. The role of osmotic pressure and ions in sperm activation and the maintenance of flagellar motility in marine teleost is still largely unknown [17]. Aquaporins may play a crucial role in inducing hyperpolarization of the sperm plasma membrane, leading to the activation of sperm motility. The impact of aquaporins on the activation of motility in marine fish sperm was investigated in gilthead sea bream [15]. When sperm are discharged into seawater, they are exposed to a high osmotic pressure environment, which results in rapid internal water exudation.

Turbot (*Scophthalmus maximus*) is a commercially significant marine flatfish species in China. Natural egg laying cannot be induced in fish farms, necessitating the artificial acquisition of sperm and eggs for artificial insemination. Given the intricate hydrodynamics involved in gamete maturation, activation, and cryopreservation, it is crucial to gain a deeper understanding of the physiological roles of aquaporins during turbot spermatogenesis. In this study, we extensively utilized available turbot genomic resources to identify 16 *aqps* genes across the genome and conducted a comprehensive phylogenetic analysis. Additionally, we investigated the expression patterns of *aqps* genes during turbot spermatogenesis and compared them with other vertebrate species to infer potential functions of *aqps* in turbot. Remarkably, our findings indicate a close relationship between *aqp1* and sperm activation.

## 2. Results

### 2.1. Identification of aqps Genes in Turbot

We used AQPs proteins from various species, including human, chicken, medaka, zebrafish, olive flounder, and black rockfish, as query sequences to identify 16 smaqps genes, which are listed in Table 1. In our study, we identified a total of 16 members of the turbot aqp family, namely aqp0a, 0b, 1, 3, 4, 7, 8aa, 8ab, 8b 9a, 9b, 10a, 10b, 11a, 11b, and 12. All the identified AQPs proteins shared a conserved domain major intrinsic protein (MIP). The length of the smAQPs proteins varied from 259 to 446 amino acid residues, with relative molecular weights (MWs) ranging from 27.07 to 48.16. Theoretical isoelectric point (PI) values spanned from 5.00 to 9.87. Predictions for subcellular localization consistently indicated that sm-AQPs proteins predominantly localize to the plasma membrane.

### 2.2. Phylogenetic Analysis of AQPs Proteins

**Figure 1:**
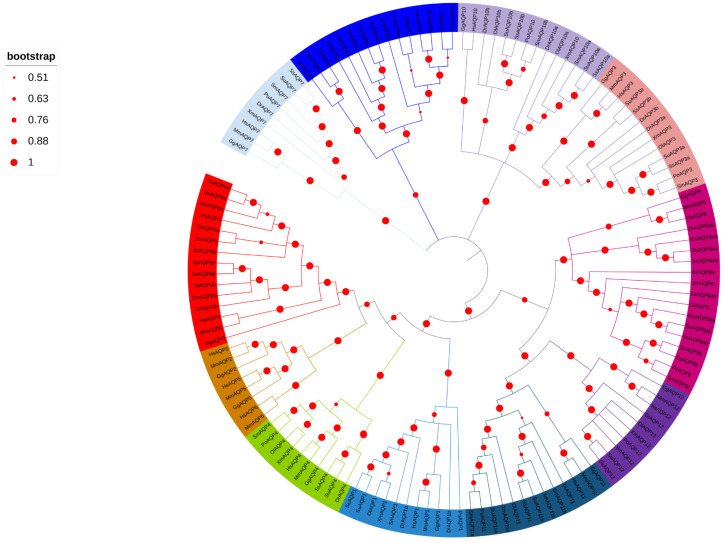
Phylogenetic analysis of AQP amino acid sequences from ten species. Each branch is color-coded to group the same AQP from different species together. The species included in the analysis were Dr (*Danio rerio*), Sm (*Scophthalmus maximus*), Po (*Paralichthys olivaceus*), Ol (*Oryzias latipes*), Su (*Sebastes umbrosus*), Xm (*Xiphophorus maculatus*), Ss (*Sebastes schlegelii*), Hs (*Homo sapiens*), Mm (*Mus musculus*), and Gg (*Gallus gallus*). The diameter of the red circles represents the size of the bootstrap on the left side. The red circles of different diameters were on the phylogenetic tree branch on the right side.

To verify the identity of *aqps* family members in turbot (Figure 1), phylogenic trees were constructed using AQP protein sequences from turbot and nine other species. The AQP protein sequences of turbot were found to exhibit a close relationship with those of olive flounder (*Paralichthys olivaceus*). Notably, *aqp2*, *5*, and *6* were absent in teleosts, but present in *Homo sapiens*, *Mus musculus*, and *Gallus gallus* exclusively.

### 2.3. Conserved Motif and Domain Analyses

**Figure 2:**
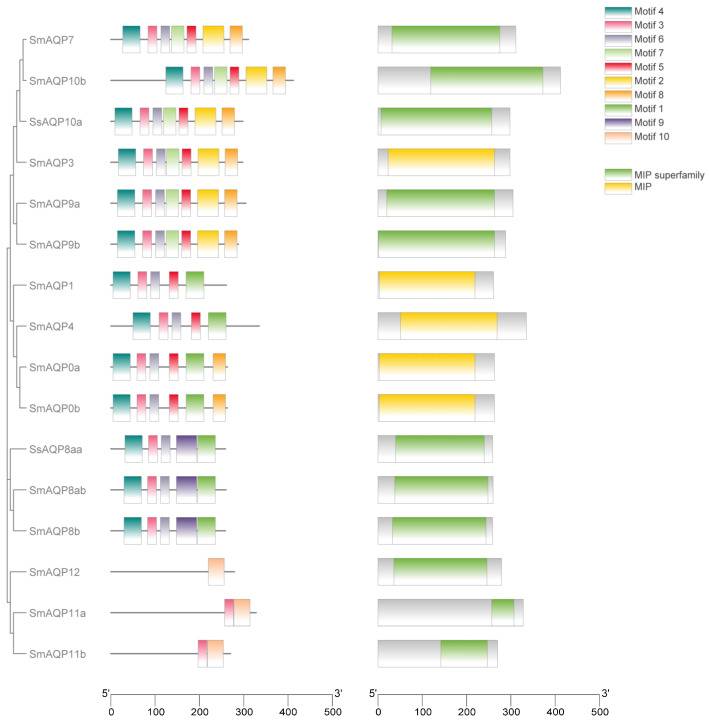
Motif and conserved domain analysis of AQP protein. The diverse domains identified were represented by different colors in the rectangles. The first column was visualization of the evolutionary tree of family members. On the left side, the colored square blocks are motifs that are conserved among AQPs (the second column). On the right side, the colored square blocks are domains that are conserved among AQPs (the third column). The fourth column is the legend: the top is motif, and the bottom is domain.

In order to explore the structural diversity of sm-*AQP*s proteins, we employed the MEME/MAST tools available online to analyze conserved motifs. Using the MEME program, we identified 10 potential protein motifs, which were subsequently subjected to motif detection through MAST (Figure 2). The gene structures among sm-AQPs were found to be highly conserved and showed high similarity with those of olive flounder. However, the structures of AQP11a, AQP11b, and AQP12 were notably different from both the other sm-AQPs genes and their homologous (Figure 2). The MIP domain was present in all AQPs members.

### 2.4. Chromosomes Localization of aqps

**Figure 3:**
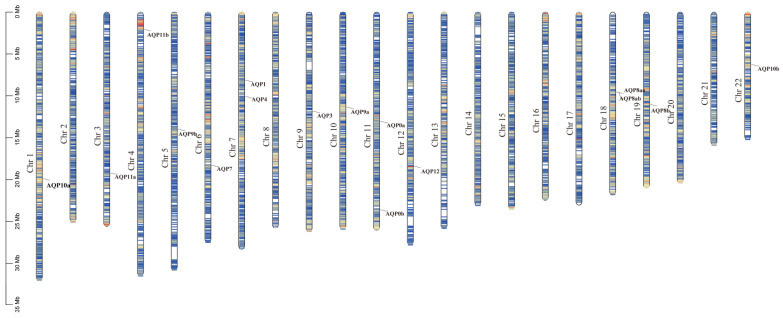
Chromosome localization of *aqp* genes in *S*. *maximus* (Chr: chromosome).

The genomic locations of aqp genes were determined using genome databases. As illustrated in Figure 3, we identified 16 aqp genes distributed across thirteen chromosomes. Specifically, aqp0a and aqp0b were located on Chr11, aqp1 and aqp4 on Chr7, aqp3 on Chr9, aqp7 on Chr6, aqp8aa and 8ab on Chr18, aqp8b on Chr19, aqp9a on Chr10, aqp9b on Chr5, aqp10a on Chr1, aqp10b on Chr22, aqp11a on Chr3, aqp11b on Chr4, and aqp12 on Chr12.

### 2.5. The Expression Patterns of aqps during Spermatogenesis

The testis development stages were classified as follows: MSII and MSIII, representing the initiation stage of the annual breeding cycle; MSIII–IV, indicating the transitional phase of the testes; MSIV, representing the spermiogenesis stage characterized by an increased proportion of spermatid; MSV, corresponding to the spawning phase; and MSVI, representing the testis recession phase.

In our previous studies, we performed RNA sequencing on testes at different developmental stages (MSII, MSIII, MSIII-IV, MSIV, MSV, MSVI) [18] and extracted all expressed and annotated members of the aqp family. During different reproductive stages, we observed high expression of aqp1, 4, 8, 10, 11, and 12 in the testis.

**Figure 4:**
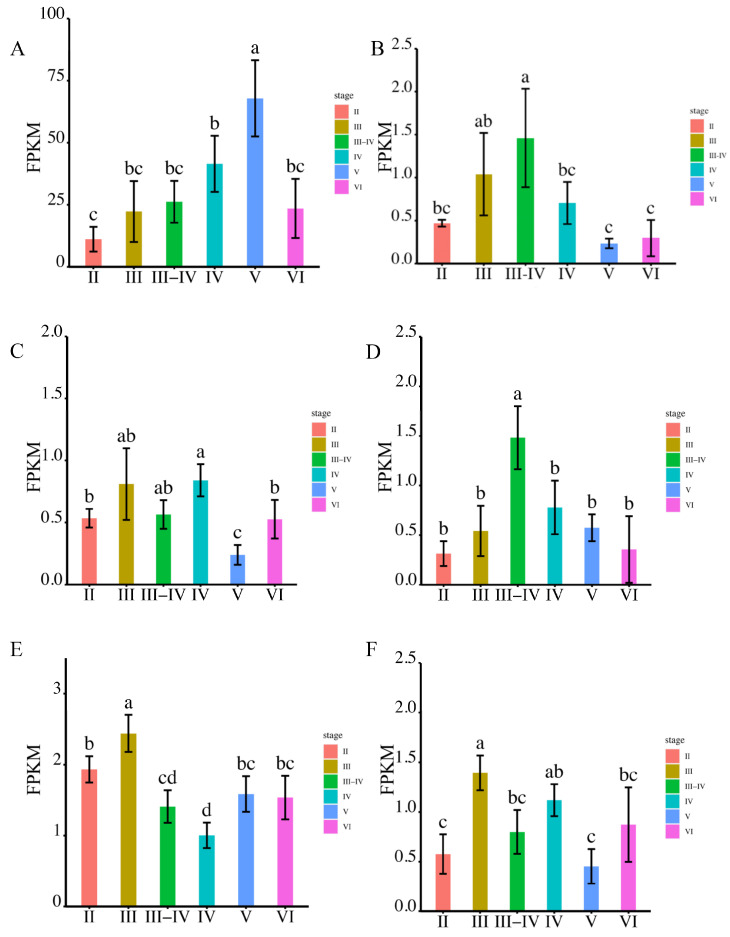
The expression levels of *aqp*s genes were analyzed using RNA-seq at various stages of testis development (*p* < 0.05). Panels (**A**–**F**) depict the expression patterns of specific aqp genes. (**A**): *aqp1*; (**B**): *aqp4*; (**C**): *aqp8*; (**D**): *aqp10*; (**E**): *aqp11*; (**F**): *aqp12*. The developmental stages analyzed include MSII and MSIII, representing the initiation stage of the annual breeding cycle; MSIII–IV, indicating the transitional phase of the testes; MSIV, representing the spermiogenesis stage with an increased proportion of spermatid; MSV, corresponding to the spawning phase; and MSVI, representing the testis recession phase. According to statistical analysis, the letter “a” represents the relative expression levels whose mean values are relatively larger. From “a” to “c”, the mean values decrease in turn. There is no significant difference between stages with the same letter. There is a significant difference between stages with different letters, (*p* < 0.05).

Among the analyzed aqps, aqp1 exhibited the highest expression during sperm maturation at the MSV stage (Figure 4A). The expression of aqp4 displayed a gradual increase from MSII to MSIII-IV, followed by a decrease (Figure 4B). Notably, there were no significant differences among MSIII, MSIII-IV, and MSIV, with all three stages demonstrating higher aqp8 expression compared to MSII, MSV, and MSVI (Figure 4C). aqp10 expression peaked at the MSIII-IV stage, without significant differences observed among the other stages (Figure 4D). Regarding aqp11, its expression was primarily detected during male germ cell proliferation at the MSII and MSIII stages, while the expression peak of aqp12 occurred during male germ cell proliferation at the MSIII and MSIV stages (Figure 4E,F).

**Figure 5:**
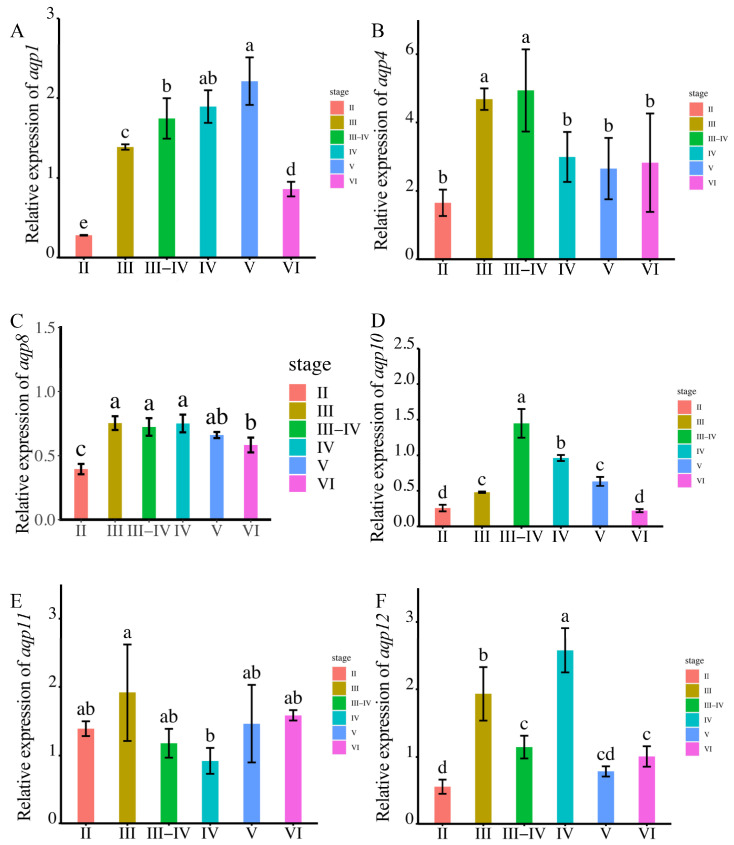
The expression levels of *aqps* genes were analyzed at different testis development stages (*p* < 0.05). The results are presented as follows: (**A**): *aqp1*; (**B**): *aqp4*; (**C**): *aqp8*; (**D**): *aqp10*; (**E**): *aqp11*; (**F**): *aqp12*. The developmental stages analyzed include MSII and MSIII, representing the initiation stage of the annual breeding cycle; MSIII–IV, indicating the transitional phase of the testes; MSIV, representing the spermiogenesis stage with an increased proportion of spermatid; MSV, corresponding to the spawning phase; and MSVI, representing the testis recession phase. The *ubq* and *rsp* genes were used as reference genes. Relative gene expression data were analyzed using the 2^−ΔΔCt^ method. All reactions were carried out in triplicate. According to statistical analysis, the letter “a” represents the relative expression levels whose mean values are relatively larger. From “a” to “d”, the mean values decrease in turn. There is no significant difference between stages with the same letter. There is a significant difference between stages with different letters, (*p* < 0.05).

To validate the observed expression trends, we conducted RT-qPCR analysis. The primers used in the present study are in Supplementary Table S1. The results demonstrated that aqp1 exhibited predominant expression during sperm maturation at the MSV stage, whereas other members of the aqp family displayed significantly higher expression levels during male germ cell proliferation stages rather than during sperm maturation (Figure 5A–F).

### 2.6. Localization of AQP1 Protein in Testis by Immunohistochemistry

**Figure 6:**
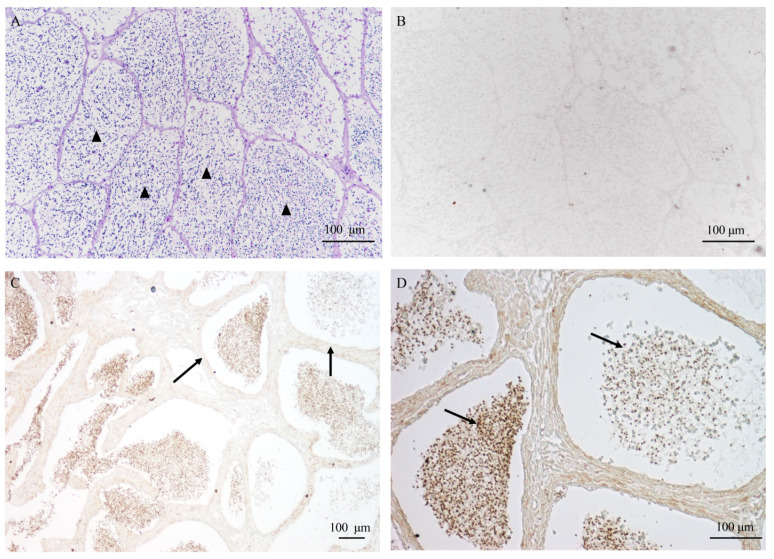
The localization of AQP1 protein in testis during sperm maturation. (**A**) the histological structure of the testis during sperm maturation, visualized through HE staining; (**B**) the negative control; (**C**) the localization of the AQP1 protein in the testis, 10×; and (**D**) the localization of the AQP1 protein in the testis, 20×. The scale bar represents 100 μm. The arrowhead in (**A**) indicates the sperm cyst; arrows in (**C**) indicate somatic cells surrounding the germ cells, and arrows in (**D**) indicate sperm exhibiting positive signal.

The AQP1 protein was identified to be approximately 29 kDa, and its localization in the testis was investigated through immunohistochemistry. In Figure 6A,B, no signal was observed in the control samples, whereas in the testis, AQP1 was predominantly located in male germ cells rather than Sertoli cells (Figure 6C). Notably, it exhibited specific localization in the head of sperm within cysts (Figure 6D), indicating a potential involvement in regulating osmotic pressure during sperm activation.

### 2.7. The Effect of AQP1 Inhibitor and Antibody on Sperm Motility

**Figure 7:**
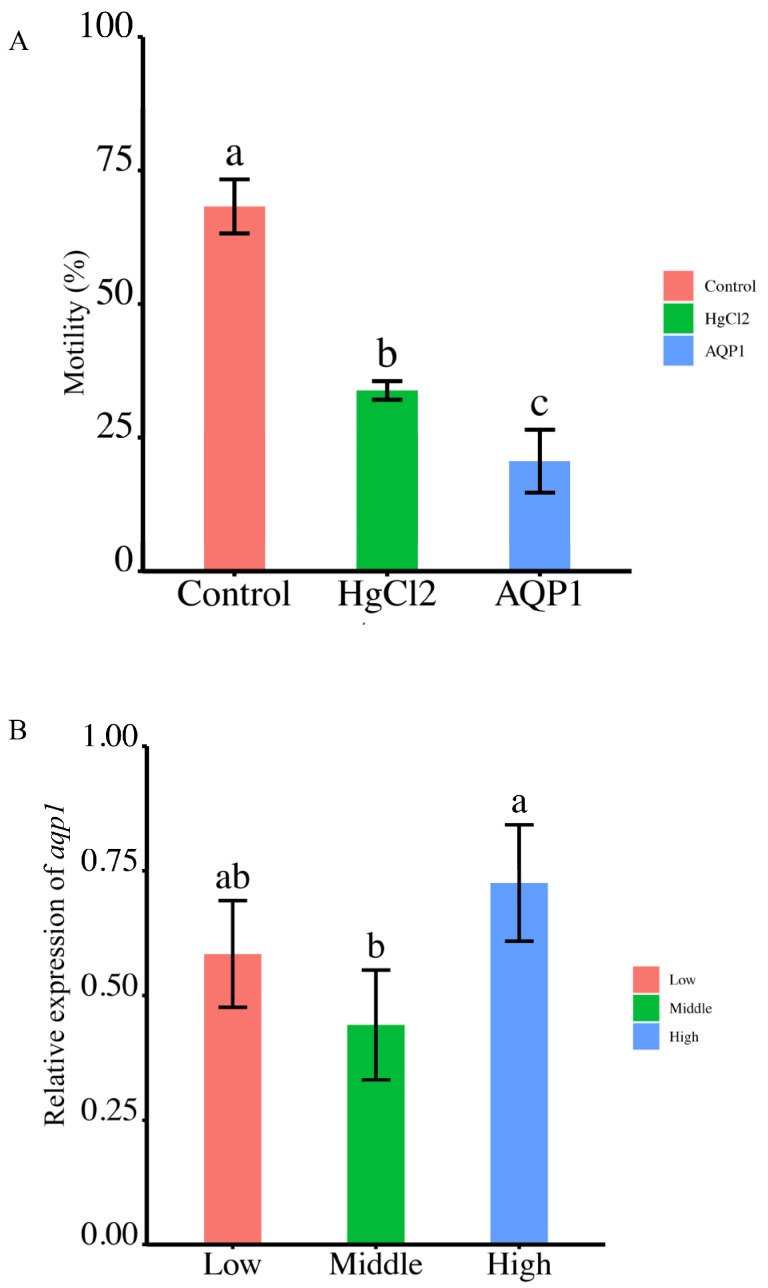
The correlation between *aqp1* expression and sperm motility. (**A**) Effects of *aqp1* inhibitor and antibody on sperm motility (*p* < 0.05). (**B**) Expression of *aqp1* across different sperm quality groups, categorized as low, middle and high sperm motility (*p* < 0.05). Motility parameters in (**A**,**B**) were motility rate. The motility range as low (<20%) to middle (40–60%) to high (80–90%) ranges in (**B**). According to statistical analysis, the letter “a” represents the relative expression levels whose mean values are relatively larger. From “a” to “c”, the mean values decrease in turn. There is no significant difference between stages with the same letter. There is a significant difference between stages with different letters, (*p* < 0.05).

Sperm motility was assessed following treatment with the AQP1-specific inhibitor, HgCl_2_, which led to a notable reduction from 68.27 ± 5.05% to 33.86 ± 1.74%. Further treatment with the AQP1 antibody resulted in a more substantial decrease to 20.57 ± 5.89% (Figure 7A). These findings demonstrate that both the AQP1-specific inhibitor and antibody significantly impaired sperm motility (Figure 7A).

### 2.8. Differential Expression of Smaqp1 RNA in Sperm of Different Quality

The expression of Smaqp1 in sperm of different quality exhibited a irregular change as motility levels escalated from low (<20%) to middle (40–60%) to high (80–90%) ranges. Notably, the highest expression was observed in the high motility group, but there were no significant differences between the low and high motility group (*p* < 0.05) (Figure 7B).

## 3. Discussion

The significantly higher copy number of aquaporins in teleosts, as compared to mammals, underscores the distinctive characteristics of *aqp* gene evolution in teleosts, making it an intriguing phenomenon. Among diploid teleosts, a singular copy of *aqp7*, *aqp12*, and *aqp14* is conserved, whereas the *aqp8* gene has undergone substantial expansion in certain species, notably the Actinopterygii fishes [19,20]. While aquaporin genes are plentiful in teleosts, it is noteworthy that *aqp2*, *aqp5*, and *aqp6* are conspicuously absent. These aquaporins have a vital function in safeguarding water balance in amphibians, sauropods, and mammals [19,21]. Consequently, it is theorized by scholars that the absence of *aqp2*, *5*, *6* in teleosts may be attributed to the evolutionary adaptation of tetrapods from sarcopterygian ancestors. Vertebrates have undergone outlet dilation and loss as a response to environmental selection pressures encountered in both aquatic and terrestrial habitats. Remarkably, the absence of *aqp2*, *5*, and *6* in turbot is noteworthy, as they are significantly more prevalent within the 0, 8, 9, 10, and 11 subfamilies, exhibiting both a and b subtypes. Compared to other teleosts, the turbot *aqp* family is relatively conservative in terms of both member number and species distribution, but it also exhibits unique characteristics.

The development, maturation, concentration, and storage of sperm rely on the metabolic process involving complex water molecules, amino acids, hormones, and other substances, with the indispensable involvement of *aqps* in germ cells and the reproductive tract. In recent years, there has been a growing recognition of the significance of aquaporins in the development of male germ cells in marine teleosts. The members of the *aqps* family exhibit diverse roles across the various stages of spermatogenesis. For example, *aqp4*, *10*, and *12* are predominantly expressed during the proliferative stage, whereas *aqp1* is primarily expressed during the mature stage. *aqp11* displays elevated expression levels during both the MSII and MSV stages, suggesting its potential significant contribution to the proliferation and maturation of male germ cells. Conversely, *aqp8* exhibits high expression levels in the MSIII, MSIII-IV, and MSIV stages, respectively, indicating a direct association with spermiogenesis. In mice, the expression of *aqp1* was observed to be the lowest, while *aqp7* exhibited the highest and abundant expression throughout all stages in the testes [22]. In humans, *aqp7* demonstrated a significant positive correlation with semen parameters [23]. *aqp11*, involved in the residual cytoplasm of spermatids, exhibited the highest expression levels compared to other organs in rats [9]. These expression patterns displayed considerable variability between species, indicating species-specific differences. In equine, *aqp4*, *7*, *8* exhibited higher transcript expression levels in the testis. *aqp7* played a significant role during spermatid cytoplasmic reduction, while *aqp8* expression was observed throughout all stages of spermatogenesis [24]. In mammals, *aqp3*, *7*, and *11* are involved in regulating sperm osmolality and cryobiology [14].

The sperm of teleost fish remain in a quiescent state within the testis and vas deferens until they are released into the water environment, at which point they become activated and initiate movement. However, the precise contribution of osmotic pressure and ions in the activation and maintenance of flagellar motility in marine teleost remains largely unexplored. In our study, we performed a correlation analysis between sperm motility and *aqp1* expression, uncovering that the *aqp* inhibitor and antibody significantly reduce sperm motility, indicating the association of *aqp1* with sperm motility. Consequently, *aqp1* is suggested to play a crucial regulatory role in the hydration and activation of turbot sperm, thereby closely influencing its motility. However, there was no direct correlation between the *aqp1* expression and sperm quality Additionally, Zilli explored the impact of aquaporin on the activation of motility in marine fish sperm. Upon release into seawater, sperm encounter a high osmotic pressure environment, leading to a rapid release internal water. This process has the potential to hyperpolarize the sperm plasma membrane and initiate sperm motility [1,15], likely involving aquaporin. Zilli employed specific AQP1 antibodies to examine the presence of a water-selective channel in both the head and tail of spermatozoa [15,25]. Treatment with HgCl_2_, a reversible aquaporin inhibitor, coupled with functional expression and sperm activation analysis, suggested the involvement of *aqp1* in mediating sperm hyperactivation in Atlantic salmon, rainbow trout [26] and gilthead sea bream [15]. The role of *aqp1* in sperm activation varies significantly between teleost fish and mammals and is possibly attributed to differences in the mechanisms of sperm activation across species in distinct environments conditions.

## 4. Materials and Methods

### 4.1. Experimental Fish, and Sample Collection

The study described in this article was approved by the ethical committee of the Institute of Oceanology, Chinese Academy of Sciences.

Turbot (1.8–3.4 kg, 40–50 cm) were reared indoors in circular tanks supplied with flow-through seawater with an ambient temperature of 18 ± 0.5 °C and a dissolved oxygen level of ≥5 mg/mL. They were fed three times each day at Shenghang Sci-tech Co., Ltd. (Weihai, China). The gonads were handled with care and divided into two lobes: one lobe was collected and fixed in 4% PFA for immunohistochemistry, while the other lobe was collected for molecular studies and flash-frozen in liquid nitrogen. Sperm samples were obtained by artificial extrusion from stage V male turbot, ensuring strict precautions were taken to prevent contamination from water, blood, urine, or feces. Each sperm sample was carefully collected using a sterile graduated pipette. Subsequently, each sample was divided into two portions: one portion was used for assessing motility, while the other portion was stored in liquid nitrogen for RNA extraction to quantify the relative expression of AQPs.

### 4.2. Phylogenetic Analysis, Gene Location on Chromosomes, and Domain Analysis

Homologous sequences of aquaporin (*aqp*) family proteins from human, chicken, medaka, zebrafish, olive flounder, and black rockfish were obtained. To obtain the turbot *aqp* family sequences, the genome database of turbot was screened using BLAST with the *aqp* family sequences as retrieval sequences. The major intrinsic protein (MIP) domain of aquaporin in each member was predicted via SMART (http://smart.embl-heidelberg.de/, accessed on 8 November 2022), enabling the identification of turbot *aqp* family members. Multisequence alignment was performed using ClustalW, and a phylogenetic tree was constructed using the MEGA11 software. The construction of the phylogenetic tree involved a comparison of AQP family protein sequences between turbot and various species, such as Dr (*Danio rerio*), Po (*Paralichthys olivaceus*), Ol (*Oryzias latipes*), Su (*Sebastes umbrosus*), Xm (*Xiphophorus maculatus*), Ss (*Sebastes schlegelii*), Hs (*Homo sapiens*), Mm (*Mus musculus*), and Gg (*Gallus gallus*). Furthermore, the structural diversity of AQP family proteins in turbot was assessed using MEME/MAST (https://meme-suite.org/meme/tools/meme, accessed on 30 November 2022).

### 4.3. RNA Seq

The cDNA libraries were constructed and subsequently sequenced. The details are described as published in reference [18]. In general, total RNA was extracted and verified for integrity using an Agilent 2200 Bioanalyzer (Santa Clara, CA, USA). Samples with RNA integrity number (RIN) ≥ 8.0 were selected for cDNA library preparation. cDNA libraries were constructed for each RNA sample, and 18 libraries for 18 samples were sequenced. Paired-end sequencing was performed using an Illumina HiSeq X-Ten (San Diego, CA, USA). Post-sequencing, raw reads were filtered to remove low-quality scores, adaptor-polluted reads, and reads with a high content of unknown bases (N). AQP gene family members identified in the data and presented at high expression levels were analyzed.

### 4.4. Real-Time PCR

Total RNA was extracted from both testis and sperm samples, with three biological samples in each group. The expression of *aqps* during spermatogenesis was analyzed using quantitative real-time polymerase chain reaction (RT-qPCR) with the SYBR Green detection method. The RT-qPCR analysis was conducted on a CFX Connect Real-time System (Applied Biosystems, BIO-RAD, Hercules, CA, USA). The *ubq* and *rsp* genes were used as reference genes. Relative gene expression data were analyzed using the 2^−ΔΔCt^ method. All reactions were carried out in triplicate.

### 4.5. Analyses of aqp1 Inhibitor and Antibody on Sperm Motility

Sperm total motility (overall proportion of sperm that are actively moving) was evaluated using a computer-assisted sperm analysis system (CASA, Tsinghua Tongfang Inc., Beijing, China). Milt was activated in natural seawater at a ratio of 1:200 (*v*/*v*), and sperm motility was measured within 30 s of activation in three visual fields. There were three samples in each group. Each group was assessed with three replicates, and each replicate was analyzed three times.

### 4.6. Immunohistochemistry Localization of AQP1 Protein in Testis

Tissue sections were dewaxed and rehydrated following standard protocols. For antigen retrieval, the sections were sequentially incubated in sodium citrate at 95 °C for 15 min, followed by washing with TBS. To block endogenous peroxidases, the sections were incubated with 3% hydrogen peroxide in methanol for 10 min at room temperature, followed by washing with TBS. Subsequently, normal goat serum (1:20; Sigma, St. Louis, MO, USA) was applied to minimize undesired background staining, and the sections were incubated for 30 min. The anti-*S. maximus* AQP1 polyclonal antibody, produced by Abclonal (Wuhan, China), was generated in rabbits using a synthetic peptide derived from the C-terminal region of AQP1 (AIGNKNNTSPDQEVKV). Subsequently, tissue sections were incubated overnight at 4 °C in a humidified chamber with primary antiserum (polyclonal rabbit anti-AQP1) diluted 1:500 in 5% goat serum (Boster Biological Company, Wuhan, China). On the subsequent day, the sections were incubated with an HRP-labeled secondary antibody (goat anti-rabbit IgG, diluted 1:500; Shanghai Biological Engineering Co., Ltd., Shanghai, China) for 1 h at room temperature. The peroxidase activity was visualized using a DAB solution, and the reaction was halted by rinsing with TBS. Negative controls were established by replacing the primary antibody with 0.01 M PBS. The results were visualized using the same methods as described above.

### 4.7. Statistical Analysis

Statistical analysis was performed using SPSS version 19.0 for Windows (SPSS Inc. Chicago, IL, USA). The data were presented as means ± SD (*p* < 0.05). The RT–qPCR data were statistically analyzed using one-way ANOVA followed by a Tukey test. All experiments were independently conducted in triplicate, and a *p* < 0.05 denoted statistically significant.

## 5. Conclusions

In the present study, we identified 16 members of the *aqp* family. Among them, *aqp1*, *4*, *8*, *10*, *11*, *12* showed abundant expression during spermatogenesis, with *aqp1* present at a particularly high expression at the sperm maturation stage. AQP1 was found to be localized in sperm and closely associated with sperm activation. These findings offer valuable insights into the *aqp* gene family’s involvement in teleost spermatogenesis. Moreover, these discoveries have the potential to improve sperm activation and cryopreservation techniques.

## Figures and Tables

**Table 1:** Physicochemical properties of *aqps*.

Gene	Number of Amino Acids	Molecular Weight (kDa)	Theoretical PI	Subcellular Location
*Smaqp0a* XP_035500823.1	263	28.681	9.36	Plasma membrane
*Smaqp0b* XP_035500169.1	261	27.457	6.41	Plasma membrane
*Smaqp1* XP_035490995.1	271	29.004	8.44	Plasma membrane
*Smaqp3* XP_035496857.1	298	32.527	7.00	Plasma membrane
*Smaqp4* XP_035491251.1	335	35.952	6.38	Plasma membrane
*Smaqp7* XP_035487470.1	311	33.349	6.08	Plasma membrane
*Smaqp8aa* XP_035469441.1	259	27.068	8.08	Plasma membrane
*Smaqp8ab* XP_035469440.1	260	27.573	6.41	Plasma membrane
*Smaqp8b* XP_035471338.1	259	27.324	7.56	Plasma membrane
*Smaqp9a* XP_035499181.1	305	32.992	5.00	Plasma membrane
*Smaqp9b* XP_035485369.1	288	30.954	6.09	Plasma membrane
*Smaqp10a* XP_035500062.1	298	32.497	6.80	Plasma membrane
*Smaqp10b* XP_035475972.1	446	48.158	8.09	Plasma membrane
*Smaqp11a* XP_035479367.2	328	35.363	9.87	Plasma membrane
*Smaqp11b* XP_035482617.2	270	28.455	8.40	Plasma membrane
*Smaqp12* XP_035503730.1	279	30.736	9.46	Plasma membrane

## Data Availability

Sequence data generated for the present study has been deposited to NCBI Short Read Archive. The SRA ID assigned is: SRP136753.

## Supplementary Materials

The following supporting information can be downloaded at: https://www.mdpi.com/article/10.3390/ijms241411770/s1.
