# Supplementary material for: Genome-Wide Identification of Aqp Family Related to Spermatogenesis in Turbot (Scophthalmus maximus)
Source: Int J Mol Sci. 2023 Jul 21;24(14):11770. doi: 10.3390/ijms241411770 (PMC10380888; doi:10.3390/ijms241411770)
Supplement: Supplementary file 1 [file ijms-24-11770-s001.zip › ijms-2491562-supplementary.pdf]

Supplementary Table S1: Primers used in the study

Primer name	Sequence	Tm
ubq	F: GCGTGGTGGCATCATTGAGC	60.5
	R: CTTCTTCTTGCGGCAGTTGACAG	59.0
rsp4	F: CAACATCTTCGTCATCGGCAAGG	58.8
	R: ATTGAACCAGCCTCAGTGTTTAGC	57.5
aqp1	F: AGGCTGTGGAATCAATCCCG	57.9
	R: GTATGGACACGTGCATTGGC	57.2
aqp4	F: CAGCCGACCTGGTCCTTATC	58.2
	R: CATTGCAGGCCCAAAGGAAC	57.6
aqp8	F: AGAACGCTACCACAATGCCA	57.0
	R: AGTAGTTGGTCATCACGGCG	57.6
aqp10	F: GATCAGAAACCGGCTGGTCA	57.7
	R: TCAGAGAAACGGCAGGGTTC	57.6
aqp11	F: GTACCGCTCGAGGATAACGG	58.0
	R: CTGCATAAACCACCGATGCG	57.2
aqp12	F: TGCGCACTCATCTTTCACCT	58.0
	R: AAGAGGAGAGCCAGGGTCAT	58.0
